# The Catheterization Laboratory: An Occupational Anachronism

**DOI:** 10.1016/j.jscai.2026.105565

**Published:** 2026-07-14

**Authors:** Alexandra J. Lansky, Dean J. Kereiakes, James A. Goldstein

**Affiliations:** a Yale School of Medicine, New Haven, Connecticut; b The Christ Hospital, Cincinnati, Ohio; c Beaumont-Corewell Health System, Royal Oak, Michigan

**Keywords:** catheterization laboratory, occupational hazard, radiation

Over the past 4 decades, cardiovascular medicine has undergone a remarkable transformation driven by relentless innovation. Catheter-based therapies have revolutionized the treatment of coronary artery disease, valvular heart disease, and peripheral vascular disease; as a result, patient outcomes have improved. Procedures that were once unimaginable are now routine in contemporary practice. However, despite these remarkable advances in patient care, the working environment for the physicians, nurses, technologists, and staff who perform these procedures in the fluoroscopic laboratories has evolved far more slowly, if at all.

Occupational hazards associated with fluoroscopically guided procedures remain substantial and largely ignored.^1^, ^2^, ^3^ Chronic radiation exposure (RE) is associated with cataracts, malignancy, and other stochastic health effects. At the same time, the physical burden of personal protective equipment (PPE), specifically heavy lead aprons required to limit RE, has contributed to high rates of orthopedic injury among interventional physicians and catheterization laboratory staff. Musculoskeletal complications, including debilitating cervical spine disease, lumbar degeneration, and other chronic orthopedic injuries, are now recognized as significant occupational consequences of contemporary interventional practice. Despite widespread adherence to established radiation safety principles, conventional protective strategies remain imperfect and outdated, leaving personnel vulnerable to both radiation-related and orthopedic injury.

As defined in Title 10, Section 20.1003 of the Code of Federal Regulations (10 CFR 20.1003), ALARA (“As Low As Reasonably Achievable”) is a federal regulatory requirement governing radiation safety. Historically, ALARA has served as the foundational framework for minimizing RE to both patients and health care personnel. However, what is “reasonably achievable” has dramatically evolved over time. The development of enhanced radiation protection devices, capable of reducing operator RE by >95% compared with conventional lead-apron–based protection, has fundamentally shifted the radiation protection paradigm.^4^ These innovations have enabled lead-free alternatives to traditional radiation protection and challenged the long-standing reliance on heavy lead aprons as the primary means of occupational shielding. Although lead-free catheterization laboratories have been implemented at select institutions and have prompted revisions of regulatory requirements in some jurisdictions,^5^ radiation safety standards, exposure limits, and PPE requirements remain inconsistent and, in most US states and regions of the world, are increasingly disconnected from contemporary evidence and available technology.

Against this backdrop, the study in the current issue of *JSCAI* by Arnold et al^6^ draws attention to another largely neglected contributor to RE: fluoroscopy system senescence. Although efforts to reduce RE rely on operator shielding, procedural technique, and radiation monitoring, the age and technological capabilities of the imaging platforms have received remarkably little scrutiny. The authors expose a concerning reality: many cardiac catheterization laboratories continue to rely on fluoroscopic systems that are effectively obsolete by contemporary standards. As a result, operators and patients may be exposed to unnecessarily higher radiation doses despite adherence to accepted radiation safety practices, raising further concern regarding institutional compliance with the ALARA principle and the adequacy of current equipment standards.

The authors surveyed 654 fluoroscopic systems across 183 institutions in Illinois and California. The findings were striking: the mean equipment age was approximately 17 years, nearly one-half of all systems was >15 years old (considered legacy), and approximately 80% of hospitals operated at least 1 legacy fluoroscopy unit. These findings expose a critical gap in contemporary radiation safety: the absence of meaningful regulatory oversight, performance benchmarks, or age-based replacement standards for fluoroscopic equipment. Although the survey was limited to 2 states, there is little reason to suspect that the problem is geographically isolated. If anything, these observations likely represent the visible portion of a much larger national and global challenge.

These observations carry important implications for both patients and health care personnel and force a reconsideration of what constitutes acceptable radiation safety in contemporary practice. Modern fluoroscopy systems incorporate substantial advances in detector technology, image processing, beam filtration, pulse management, and dose optimization, enabling superior image quality while substantially reducing RE. Collectively, these innovations are associated with reductions in RE of approximately 30% to 60% while improving image quality,^7^ underscoring that continued reliance on aging fluoroscopy systems subjects both patients and operators to unnecessarily higher radiation doses while providing inferior imaging performance. The ALARA principle requires that RE be reduced to the lowest level reasonably achievable; however, what is reasonably achievable today bears little resemblance to what was achievable when many of these fluoroscopy systems were installed. In this context, continued reliance on obsolete imaging platforms is fundamentally inconsistent with contemporary interpretations of ALARA and warrants careful scrutiny from both institutional and regulatory perspectives. Fluoroscopy infrastructure should therefore be viewed not merely as an engineering or capital-planning issue, but as a critical component of patient safety and occupational health. In an era when technologies capable of dramatically reducing RE are readily available, failure to modernize aging fluoroscopy infrastructure perpetuates avoidable harm and demands urgent attention.

The observations of Arnold et al are particularly timely when viewed alongside the recent multisociety ALARA+ Summit and accompanying consensus statement,^6^ endorsed by major cardiovascular, radiologic, and neurovascular societies, which characterized protection of fluoroscopy laboratory personnel as a “moral imperative.”^8^ Together, these efforts signal an important evolution in how occupational safety should be viewed within procedural medicine. The implications extend beyond RE alone. Historically, discussions surrounding occupational safety have focused on balancing radiation reduction against the ergonomic burden of lead-based PPE. The profound reduction in “achievable” RE by enhanced radiation protection devices has fundamentally changed that equation. When combined with contemporary fluoroscopy platforms designed to minimize radiation generation at the source, the opportunity now exists to simultaneously reduce RE and lessen dependence on heavy lead apparel. The goal should no longer be mitigation of occupational risk; it should be elimination of preventable occupational risk.

Perhaps the most important message emerging from both the study by Arnold et al and the ALARA+ initiative is that radiation safety can no longer be viewed solely through the lens of individual operator behavior. RE is increasingly a systems-level issue encompassing imaging infrastructure, shielding technologies, institutional investment, regulatory oversight, and workplace culture. Reliance on aging fluoroscopy equipment while expecting operators to compensate through hazardous PPE alone is inconsistent with contemporary standards of occupational safety. The catheterization laboratory has long served as a cradle of cardiovascular therapeutic innovations. However, the widespread persistence of aging fluoroscopy infrastructure suggests that, in at least one important respect, many laboratories have become technological anachronisms. The data presented by Arnold et al should prompt a critical reassessment of how institutions define and achieve radiation safety. If enhanced protection technologies and modern imaging platforms are available and are demonstrably superior, continued reliance on outdated systems becomes increasingly unjustifiable.

The challenge now extends beyond recognition to action. Professional societies, hospital systems, regulatory agencies, and industry partners must work collaboratively to establish evidence-based standards for fluoroscopy infrastructure, radiation protection technologies, and equipment replacement. The goal should not be mere compliance with historical interpretations of ALARA, but the adoption of contemporary standards that reflect what is now achievable. Institutions should not be permitted to satisfy radiation safety obligations through operator-dependent mitigation strategies alone while continuing to rely on aging imaging platforms that generate unnecessary exposure at the source.

The era in which occupational RE and orthopedic injury were accepted as unavoidable consequences of interventional practice must come to an end. Technologies now exist that can dramatically reduce and virtually eliminate both hazards. The question is no longer whether we can create a safer catheterization laboratory, but whether we have the collective will to make it the standard of care.

## CRediT authorship contribution statement

**Alexandra J. Lansky:** Conceptualization, Writing – original draft. **Dean J. Kereiakes:** Conceptualization, Writing – review & editing. **James A. Goldstein:** Conceptualization, Writing – review & editing.

## Peer review statement

Editor in Chief Alexandra J. Lansky, Deputy Editor Dean J. Kereiakes, and Associate Editor James A. Goldstein had no involvement in the peer review of this article and has no access to information regarding its peer review. Full responsibility for the editorial process for this article was delegated to Deputy Editor Suzanne J. Baron.

## Declaration of competing interest

Dean Kereiakes owns equity in Egg Medical, Inc, a manufacturer of enhanced radiation protection devices equipment. James Goldstein owns equity and serves on the board of ECLS, Inc, a manufacturer of enhanced radiation protection devices equipment. Alexandra Lansky declares no conflict of interest.
